# Breakfast skipping alone and in interaction with inflammatory based quality of diet increases the risk of higher scores of psychological problems profile in a large sample of Iranian adults

**DOI:** 10.1017/jns.2020.62

**Published:** 2021-02-16

**Authors:** Fahimeh Haghighatdoost, Awat Feizi, Ahmad Esmaillzadeh, Ammar Hassanzadeh Keshteli, Hamid Afshar, Peyman Adibi

**Affiliations:** 1 Isfahan Cardiovascular Research Center, Cardiovascular Research Institute, Isfahan University of Medical Sciences, Isfahan, Iran; 2 Psychosomatic Research Center, Isfahan University of Medical Sciences, Isfahan, Iran; 3 Biostatistics and Epidemiology Department, School of Health and Psychosomatic Research Center, Isfahan University of Medical Sciences, Isfahan, Iran; 4 Gastroenterology and Hepatology Research Center, Isfahan University of Medical Sciences, Isfahan, Iran; 5 Department of Community Nutrition, School of Nutritional Sciences and Dietetics, Tehran University of Medical Sciences, Tehran, Iran; 6 Department of Medicine, University of Alberta, Edmonton, Alberta, Canada

**Keywords:** Breakfast skipping, Dietary inflammatory index, Depression: Anxiety, Distress, Psychological problems

## Abstract

The authors investigate the association of breakfast skipping and its interaction with a dietary inflammatory index (DII) with the severity of psychological disorders. A total of 2876 Iranian general adults were enrolled in this cross-sectional study. Psychological problems profile score was calculated using the regression method in the framework of factor analysis based on depression, anxiety and psychological distress. The higher scores indicate more severity of mental problem. The frequency of breakfast eating in a week was assessed. Dietary intakes were assessed using a food-frequency questionnaire and twenty-seven items were included in the calculation of DII. In the crude model, individuals who ate breakfast seldom had the highest odds for having worse psychological problems profile (OR 3⋅59; 95 % CI 2⋅52, 5⋅11). Adjustment for various confounders did not change the associations (OR 3⋅35; 95 % CI 2⋅11, 5⋅32). In the adjusted multinomial logistic regression model, participants with high DII (>median) who skipped breakfast had highest risk of being in the higher tertiles of psychological problems profile compared with those who had low DII (<median) and ate breakfast (OR 6⋅67; 95 % CI 3⋅45, 12⋅90). Similar results were observed in women and men regarding the impact of breakfast skipping alone and interaction with DII on scores of psychological problems profile. Breakfast skipping is associated with higher risk of psychological problems. Similar findings were obtained in the stratified analysis by sex. Our findings confirmed that the DII and breakfast skipping are associated with mental health, interactionally. Further longitudinal studies are needed to confirm the true link between breakfast skipping and psychological problems.

## Introduction

Based on the World Health Organization reports, the burden of mental disorders will increase to 15 % 2020^(1)^. Although there are many possible efficient ways to treat mental disorders, half of all Americans who suffer from a severe mental disorder do not receive treatment^(2)^. In addition, due to the direct link between mental illness and metabolic disorders^(3)^, taking some actions to prevent is more helpful rather than treating. Accordingly, the identification of some relevant lifestyle-related factors may be necessary in this context.

There is evidence that unhealthy eating habits, such as breakfast or dinner skipping and irregular meal patterns may affect metabolic profile^(4–7)^, brain activity^(8–10)^ and mental health status^(11–16)^. However, the association between breakfast skipping and mental health has been rarely investigated and yielded conflicting evidence. While some investigations have found a direct link between breakfast skipping and the risk of psychological problems^(11,13,16–19)^, some others failed to find any link. For example, an investigation among adolescents indicated that only a high-quality breakfast, containing at least three food groups, was associated with better mental health^(15)^. A 2-year prospective cohort study among Japanese adults^(12)^ and a cross-sectional study in haemodialysis patients^(14)^ found no relationship between breakfast skipping and depressive symptoms. Differences in study populations (age, sex, socioeconomic status, lifestyle and consumed foods especially in breakfast) may explain the conflicting findings.

Breakfast is specifically defined as ‘the first meal of the day that breaks the fast after the longest period of sleep and is consumed within 2 to 3 h of waking; it is comprised of food or beverage from at least one food group and may be consumed at any location’^(20)^. Although breakfast consumption is known as a health-promotion dietary behaviour, skipping breakfast is a common dietary habit in both adults^(21)^ and adolescents^(22)^, and it is more frequent in socioeconomically disadvantaged groups^(23)^. Breakfast consumption may promote mental health by lowering insulin resistance^(24)^ and cortisol production^(25)^ which affect moods^(3)^. In addition, it is possible that higher diet quality and nutrient intakes^(26)^, particularly those involved in neurons function (e.g. pyridoxine and omega-3 fatty acids) besides other healthy lifestyle behaviours such as not smoking, less drinking alcohol and higher physical activity levels^(16,27,28)^ in breakfast consumers favourably affect psychological well-being. In this regard, various bioactive components in the diet may exert pro- or anti-inflammatory effects. Therefore, pro-inflammatory features of an unhealthy diet may underpin chronic inflammation and psychological disorders^(29)^. Several investigations have evaluated the relationship between the dietary inflammatory index (DII), reflective of the potential pro-inflammatory properties of a diet, with psychological disorders and mostly indicated a direct link between DII and the risk of psychological disorders^(30–37)^.

Given that lifestyle differs by sociocultural factors, epidemiological studies in different populations can provide new insight into the relationship between dietary behaviours (i.e. breakfast eating) and different health outcomes such as mental health. Although earlier studies have controlled their results for various lifestyle-related confounders, they have mainly conducted in high-income countries and most of them have not assessed the quality of diet between breakfast eaters and skippers as well as their interaction. Therefore, we conducted a cross-sectional study to examine the association between breakfast skipping and the risk of higher scores of psychological problems profile (a combined variable based on three common mental problems, i.e. depression, anxiety and psychological distress) in nonacademic staff of Isfahan University of Medical Sciences (IUMS) with different socioeconomic levels like a general population of Iranian adults, where due to being a low-income country, breakfast might be skipped more frequently. Moreover, we evaluate the role of diet quality, determined by the inflammatory potential of diet, in interaction with breakfast skipping on severity of psychological disorders.

## Methods and materials

### Participants

The Epidemiology of Psychological, Alimentary Health and Nutrition (SEPAHAN) project is a cross-sectional study which conducted in 2010 among nonacademic staff of Isfahan University of Medical Sciences (IUMS) with different socioeconomic status who were working in fifty different centres across Isfahan province. The majority of participants were involved in administrative tasks in IUMS, rather than health provider systems, and some university hospitals and research centres were excluded because the heavy workload might decrease their collaborative rate. The participants were selected using a multistage cluster and convenience sampling method. The main aim of SEPAHAN project was to evaluate the relation of common gastrointestinal disorders with lifestyle factors and psychological disorders. More details regarding SEPAHAN project have been provided elsewhere^(38)^. Data collection was carried out in two separate consecutive phases to enhance the accuracy and response rate. In the first phase, performed in April 2010, 10 087 self-administered questionnaires regarding demographic information, anthropometric measurements and dietary intakes were distributed. The response rate in this phase was 86⋅16 % and 8691 completed questionnaires were returned. Information regarding gastrointestinal symptoms and psychological disorders were collected in the second phase (in mid-May 2010) using self-administered valid questionnaires. In the second phase, 5614 completed questionnaires out of 8691 distributed questionnaires were returned (response rate: 64⋅6 %). Finally, 4763 completed questionnaires gathered in the two phases could be matched. In the present study, 2876 persons who had complete information regarding both the frequency of breakfast consumption and psychological problems, i.e. depression, anxiety and distress, were included in the statistical analysis.

### Assessment of the frequency of breakfast consumption

The frequency of breakfast eating was assessed by asking ‘On how many days in a week do you usually eat breakfast?’. Possible answers were classified into four categories: (1) never or 1 d/week, (2) 2–4 d/week, (3) 5–6 d/week and (4) every day. In the present study, using the aforementioned responses, we categorised participants into three groups for the frequency of breakfast consumption: (1) seldom (who ate breakfast never or 1 d/week), (2) sometimes (who ate breakfast 2–4 d/week) and (3) always (who ate breakfast 5 or more d/week).

### Assessment of psychological problems and psychological problems profile

In SEPAHAN, information about psychological problems, including depression, anxiety and psychological distress, was obtained from validated Iranian version of Hospital Anxiety and Depression Scale (HADS)^(39)^ and General Health Questionnaire (GHQ-12)^(40)^, respectively. HADS includes two separate sections which measure the severity of anxiety and depression. There are 7 items with a four-point rating scale in each section. Higher scores indicate a greater degree of anxiety or depression. The possible score ranged from 0 to 21 for both disorders. Scores of 8 or higher on either section were considered to indicate the presence of anxiety or depression, and scores of 7 or less were considered normal. Cronbach's alpha coefficient (to test reliability) has been found to be 0⋅78 for the HADS anxiety sub-scale and 0⋅86 for the HADS depression sub-scale. Known groups validity of HADS showed satisfactory results; in which both anxiety and depression sub-scales showed good discrimination between different subgroups of patients in clinical settings as defined by their disease stage^(39)^.

Reliability analysis of GHQ-12 in the Iranian general population showed satisfactory internal consistency (Cronbach's alpha coefficient 0⋅87). Convergent validity indicated a significant negative correlation between the GHQ-12 and global quality of life scores (*r* −0⋅56; *P* < 0⋅0001). Also, the two-factor structure has been extracted by factor analysis that jointly accounted for 51 % of the variance of 12-main questions. In the GHQ, there are 12 questions regarding psychological distress^(40)^. A four-point rating scale has been provided for each question as follows: less than usual, no more than usual, rather more than usual or much more than usual. In accordance with the original Persian version of GHQ that revealed the bimodal scoring method (0-0-1-1) was useful in the Iranian population, the scores of distress were calculated by bimodal scoring fashion. Accordingly, the first two answers (less than usual and no more than usual) were scored ‘0’, and the second two answers (rather more than usual or much more than usual) were scored ‘1’. The maximum score could be 12 and higher scores demonstrate an elevated level of distress. In the present study, participants were considered to have psychological distress if they scored ≥4.

In this analysis, we used factor analysis for constructing a combined variable representing the overall and comprehensive assessment of the status of psychological problems. By this way, ‘psychological problems profile’ loaded by all three mental health disorders (loadings were 0⋅91 for anxiety, 0⋅90 for depression and 0⋅86 for psychological distress) was obtained. The scores of identified factors were calculated using the regression method in the framework of factor analysis. In our proposed model, ‘psychological problems profile’ as a latent response was regressed on breakfast consumption as a predictor that provides a comprehensive and integrative examination of the relation between two variables. The higher scores of psychological problems profile present more severity of psychological problems. The tertiles of factor scores were used in the association analyses.

### Assessment of dietary intake

Dietary intake was assessed by using a validated, self-administered 106-item dish-based food-frequency questionnaire (DFQ). The DFQ was designed according to the Willet-format food-frequency questionnaire to assess dietary intakes over the preceding year. More information about the design, validity and reliability of DFQ has been described elsewhere^(41)^. Briefly, food items were categorised into five main groups: mixed dishes (cooked or canned, *n* 29); grain-based foods and potatoes (*n* 10); dairy products (milk and dairy products, including butter and cream, *n* 9); fruit and vegetables (*n* 22) and miscellaneous food items and beverages (including sweets, fast foods, nuts, desserts and beverages, *n* 36). For each item, the most common portion sizes familiar to all people were asked to determine the amount of food consumed. Nine different levels, from ‘never or less than once a month’ to ‘12 or more times per day’ based on the popularity of food items, were considered to indicate the frequency of consumption. For example, for foods that are consumed less frequently, high-frequency items were omitted but kept for foods that are consumed regularly. Household measures were used to convert food items to g/d^(42)^. Daily energy and nutrient intakes of each participant were estimated based on the USDA food composition database^(43)^.

### Calculation of DII

DII, as a measure of diet quality, was calculated according to the method suggested by Shivappa *et al.*^(44)^. Accordingly, first, the energy-adjusted dietary components through the residual method were computed^(45)^. Then, each dietary component was linked to its regionally representative database that indicates an estimate of global mean intake for each item, along with its standard deviation provided in the DII definition^(44)^. Next, given that standard means were subtracted from these values and divide by the corresponding standard deviation. The *z* scores derived in this way were converted to percentile and then doubled and ‘1’ was subtracted. Finally, the centred percentiles for each parameter as multiplied by its corresponding overall inflammatory effect score, and all derived values were summed to create the overall DII. In the present study, since some components of DII had not been assessed in the DFQ or not usually consumed in Iranians’ diet as a Muslim population (like ethanol), we did not include them in the DII calculation. Therefore, the DII was calculated based on 27 nutrients, onions, tea and caffeine, which derived from the DFQ in the present study.

### Assessment of covariates

The general characteristics of participants including sex, age, marital status (single, married, divorced and widowed), educational level [duration of education (years)] and smoking habit (non-smoker, former smoker and current smoker) were assessed using a pre-tested self-administered questionnaire. The current level of participants’ physical activity was assessed using General Practice Physical Activity Questionnaire^(46)^ and participants were categorised as moderately active (1–3 h/week), active (>3 h/week), moderately inactive (<1 h/week) and inactive (no physical activity). The consumption of psychotropic medicines including nortriptyline, amitriptyline, imipramine, fluoxetine, citalopram and fluvoxamine during the last 6 months was assessed. The consumption of at least one of these medicines was taken into account as a psychotropic consumer. Because of the close relation between gastrointestinal disorders and psychological health^(47)^, we considered functional gastrointestinal disorders (FGIDs) as an important covariate in our analysis. Suffering from gastrointestinal disorders was assessed using a valid and modified Iranian version of ROME III questionnaire^(48)^. FGID was defined as suffering from at least one of the following main gastrointestinal disorders: gastroesophageal reflux, dyspepsia, irritable bowel syndrome, bloating and constipation. Body mass index (BMI) was calculated as weight (kg) divided by height squared (m^2^).

### Statistical analysis

Participants were categorised into three groups based on the frequency of breakfast consumption (seldom: never or 1 d/week, sometimes: 2–4 d/week and always: ≥5 d/week) as well as the tertiles of psychological problems profile's scores. To determine significant differences in demographic characteristics across the categories of frequency consumption and tertiles of psychological problems profile, we used analysis of variance (ANOVA) and *χ*^2^ test for continuous and categorical variables, respectively. Pairwise comparisons between groups were conducted both in ANOVA and *χ*^2^ tests, and Bonferroni adjustment was adopted. Age- and sex-adjusted means for energy intake, and age-, sex- and energy-adjusted means for other dietary variables were compared across the categories of breakfast consuming frequency by analysis of covariance (ANCOVA). We used multinomial logistic regression to estimate odds ratios (ORs) (95 % confidence interval) for having higher scores of psychological problems profile by decreasing the frequency of breakfast eating in crude and multivariable-adjusted models. In model 1, adjustment was made for demographic variables including age, sex, marital status and educational level. Model 2 was additionally adjusted for lifestyle factors which may be related to mental disorders including BMI^(49)^, smoking^(50)^ and physical activity^(51,52)^. Additional adjustment in model 3 was conducted for FGIDs and psychotropic medicines. Due to the potential effect of breakfast consumption on diet quality^(26)^ as well as the close relation between some nutrients and mental health status^(53)^, adjustment for dietary intakes of magnesium, riboflavin, pyridoxine, folate, cobalamin, Docosahexaenoic acid and Eicosapentaenoic acid, energy, fibre and caffeine was made in model 4. Stratified analyses by sex, applying the above-mentioned models, were run to examine the association of breakfast skipping and breakfast skipping/DII interaction with psychological problems profile scores separately in men and women. *P* for linear trends was determined by linear by linear *χ*^2^ test. Statistical significance (two-sided) was accepted at *P* < 0⋅05.

## Results

The mean age of study participants was 36⋅3 years (ranged from 19 to 70 years) and 1960 were females. The participants’ characteristics across the frequency of breakfast consumption are shown in Table 1. Of the total study population, 78⋅1 % of participants (*n* 2247) always consumed breakfast, 14⋅7 % (*n* 424) sometimes ate breakfast and 7⋅13 % (*n* 205) seldom consumed breakfast. Compared with individuals who seldom or sometimes consumed breakfast, individuals in the always category had higher mean age (*P* < 0⋅0001) and were more likely to be male (*P* = 0⋅001). However, the prevalence of depression or anxiety or psychological distress (*P* < 0⋅0001), psychotropic medicine use (*P* < 0⋅0001), FGIDs totally (*P* < 0⋅0001) and most of evaluated gastrointestinal disorders were lower in those who always consumed breakfast compared with those in the seldom and sometimes categories. Also, the prevalence of consuming higher pro-inflammatory diet was significantly higher among people who did not consume breakfast (*P* = 0⋅002). The general characteristics across the categories of breakfast consumption frequency by sex are also shown in Supplementary Table S1 of Supplementary material. Such as total population, the distribution of the majority of confounders was significantly different between the categories of breakfast consumption both in men and women and the distribution of these confounders was not similar in men and women; accordingly, we conducted a stratified analysis by gender for follow up the present study main objectives.

**Table 1.** General characteristics of participants across the categories of breakfast consuming frequency^a^

Variables	Breakfast consuming frequency			*P* value^b^
	Seldom	Sometimes	Always	
Participants (*n*)	205	424	2247	–
Age (years)^c^	35⋅02 ± 0⋅53	34⋅98 ± 0⋅37	36⋅58 ± 0⋅17^‡^^,^^§^	<0⋅0001
BMI (kg/m^2^)^c^	24⋅85 ± 0⋅26	25⋅10 ± 0⋅20^‡^	24⋅85 ± 0⋅08^§^	<0⋅0001
Depressed (%)	44⋅7	36⋅3	25⋅4	<0⋅0001
Anxious (%)	22⋅7	20⋅7	11⋅2	<0⋅0001
Psychologically distressed (%)	39⋅8	30⋅7	20⋅3	<0⋅0001
Male (%)	32⋅4	37⋅6	43⋅5	0⋅001
Marital status (%)				0⋅808
Married	79⋅8	82⋅9	81⋅5	
Single	18⋅9	15⋅3	16⋅9	
Other	1⋅3	1⋅8	1⋅6	
Psychotropic medicines (%)	13⋅0	6⋅6	4⋅8	<0⋅0001
Current smokers (%)	13⋅9	15⋅4	13⋅5	0⋅527
Physical activity >1 h/week (%)	9⋅8	12⋅7	13⋅3	0⋅144
Educational level				0⋅409
<12 years	15⋅9	12⋅3	11⋅4	
12–16 years	76⋅3	80⋅9	80⋅8	
≥16 years	7⋅8	6⋅8	7⋅7	
FGID (%)^d^	62⋅2	55⋅0	49⋅1	<0⋅0001
IBS (%)	26⋅1	24⋅2	21⋅4	0⋅07
Functional dyspepsia (%)	21⋅0	16⋅5	13⋅2	0⋅003
Gastroesophageal Reflux Disease (%)	33⋅2	27⋅1	21⋅1	<0⋅0001
Functional bloating (%)	16⋅4	22⋅3	20⋅5	0⋅24
Constipation (%)	29⋅8	26⋅9	22⋅2	0⋅007
High DII (More than median of DII score) (%)	58⋅3	51⋅3	48⋅6	0⋅002

BMI, body mass index; FGID, functional gastrointestinal disorders; IBS, irritable bowel syndrome; DII, dietary inflammatory index.

a Eating breakfast frequency was defined as seldom: never or 1 d/week, sometimes: 2–4 d/week and always: ≥5 d/week.

b Derived from one-way ANOVA and *χ*^2^ test for continuous and categorical variables, respectively.

c Values are means ± se.

d FGID defined as suffering from at least one of the following disorders: gastroesophageal reflux, dyspepsia, irritable bowel syndrome and constipation.

‡ Different from seldom.

§ Different from sometimes.

The general characteristics of participants across the tertiles of psychological problems profile scores are presented in Table 2. Participants in the higher tertiles of psychological problems profile had significantly greater scores for depression, anxiety and psychological distress than those in the first tertile. The proportion of male in the highest tertile was significantly lower than the first tertile (T3 29⋅8 % *v*. T1 51⋅9 %; *P* < 0⋅0001). The proportion of married subjects was slightly different across the tertiles of psychological problems profile scores (T3 80⋅6 %, T2 82⋅7 % and T1 80⋅7 %; *P* = 0⋅023). Individuals who were in the highest tertile of psychological problems profile scores were less educated (*P* < 0⋅0001) and less physically active (T1 10⋅7 *v*. T3 14⋅7; *P* = 0⋅018), but more probable to be smoker (T3 15⋅9 % *v*. T1 11⋅9 %; *P* = 0⋅026) or have FGID (T1 72⋅8 % *v*. T3 29⋅3 %; *P* < 0⋅0001) or consume psychotropic medicines (T3 21⋅8 % *v*. T1 3⋅9 %; *P* < 0⋅0001). The proportion of overweight or obese participants was not statistically different (T3 46⋅8 %, T2 45⋅0 % and T1 36⋅2 %; *P* = 0⋅32). We also evaluated the distribution of confounders across the categories of psychological problems profile scores separately in women and men and similar results with total population were obtained, although the distribution of these confounders was not the same in women and men; suggesting stratified analyses by gender for follow up the present study main objectives (Supplementary Table S2 of Supplementary material).

**Table 2.** General characteristics across the tertiles of psychological problems profile scores^a^

Variables	Tertiles of psychological problems profile scores			*P* value^b^
	1 (*n* 958)	2 (*n* 959)	3 (*n* 959)	
Age (years)	35⋅89 ± 0⋅25	36⋅62 ± 0⋅26^‡^	35⋅83 ± 0⋅24^§^	0⋅045
BMI (kg/m^2^)	24⋅91 ± 0⋅11	24⋅82 ± 0⋅12	24⋅82 ± 0⋅13	0⋅828
Depression score	3⋅13 ± 0⋅04	5⋅67 ± 0⋅04^‡^	9⋅5 ± 0⋅09^‡^^,^^§^	<0⋅0001
Anxiety score	0⋅66 ± 0⋅03	2⋅59 ± 0⋅05^‡^	7⋅28 ± 0⋅11^‡^^,^^§^	<0⋅0001
Psychological distress score	0⋅2 ± 0⋅01	1⋅20 ± 0⋅04^‡^	4⋅80 ± 0⋅09^‡^^,^^§^	<0⋅0001
Male (%)	51⋅9	41⋅8	29⋅8	<0⋅0001
Married (%)	80⋅7	82⋅7	80⋅6	0⋅023
Educational level (%)				<0⋅0001
≤12 years	35⋅7	38⋅6	42⋅6	
12–16 years	55⋅1	54⋅4	51⋅2	
>16 years	9⋅2	6⋅9	6⋅2	
Physical activity >1 h/week (%)	14⋅7	12⋅9	10⋅7	0⋅018
Psychotropic medicines use^c^ (%)	3⋅9	6⋅1	21⋅8	<0⋅0001
FGID^d^ (yes) (%)	29⋅3	51⋅5	72⋅8	<0⋅0001
IBS (%)	10⋅3	20⋅8	36⋅0	<0⋅0001
Functional dyspepsia (%)	3⋅7	13⋅0	27⋅3	<0⋅0001
Gastroesophageal Reflux Disease (%)	13⋅1	21⋅8	36⋅2	<0⋅0001
Functional bloating (%)	20⋅6	24⋅2	17⋅7	0⋅001
Constipation (%)	13⋅1	25⋅2	33⋅9	<0⋅0001
Breakfast skippers (%)	4⋅3	5⋅9	12⋅4	<0⋅0001
High DII (More than median of DII score) (%)	45⋅4	49⋅3	56⋅1	<0⋅0001
Current smokers (%)	11⋅9	13⋅5	15⋅9	0⋅026
Overweight or obese^d^ (%)	46⋅2	45⋅0	46⋅7	0⋅332
DII and breakfast eating habit				<0⋅0001
Low DII and eat breakfast (%)	52⋅5	47⋅7	38⋅8	
High DII and eat breakfast (%)	43⋅2	46⋅2	48⋅8	
Low DII and skip breakfast (%)	2⋅0	2⋅8	4⋅4	
High DII and skip breakfast (%)	2⋅3	3⋅1	8⋅0	

BMI, body mass index; FGID, functional gastrointestinal disorders; IBS, irritable bowel syndrome; DII, dietary inflammatory index.

a Values are mean ± se unless otherwise indicated.

b Resulted from one-way ANOVA and *χ*^2^ test for continuous and categorical variables, respectively.

c FGID defined as suffering from at least one of the following gastrointestinal disorders: gastroesophageal reflux, dyspepsia, irritable bowel syndrome and constipation. Overweight was defined as BMI greater than or equal to 25 and less than or equal to 29⋅99 kg/m^2^ and obese was defined as BMI ≥ 30 kg/m^2^.

‡ Different from seldom.

§ Different from sometimes.

The age-, sex- and energy-adjusted dietary intakes of participants across the categories of breakfast consuming frequency are presented in Table 3. Individuals who ate breakfast for ≥5 d/week had greater intake of carbohydrate (*P* = 0⋅006), thiamin (*P* = 0⋅038), riboflavin and long-chain omega-3 fatty acids (*P* < 0⋅0001), fruit (*P* = 0⋅005) and red meats (*P* = 0⋅007), but lower intake of fat and caffeine (*P* = 0⋅002). Dietary intakes of protein, magnesium, pyridoxine, folate and cobalamin were not different across the categories of breakfast consuming frequency.

**Table 3.** Dietary intakes of participants across the categories of breakfast consuming frequency^a^

Nutrients	Breakfast consuming frequency			*P* value^b^
	Seldom (*n* 205)	Sometimes (*n* 424)	Always (*n* 2247)	
Energy (kcal/d)	2298⋅80 ± 56⋅91	2285⋅87 ± 39⋅58	2424⋅24 ± 17⋅17	0⋅001
Protein (g/d)	88⋅87 ± 0⋅96	88⋅33 ± 0⋅66	88⋅78 ± 0⋅29	0⋅816
Fat (g/d)	101⋅11 ± 1⋅29	101⋅18 ± 0⋅90	98⋅23 ± 0⋅39	0⋅002
Carbohydrate (g/d)	290⋅19 ± 3⋅37	290⋅73 ± 2⋅35	297⋅57 ± 1⋅02	0⋅006
Magnesium (mg/d)	327⋅21 ± 3⋅82	324⋅12 ± 2⋅66	330⋅93 ± 1⋅15	0⋅052
Thiamin (mg/d)	1⋅80 ± 0⋅04	1⋅83 ± 0⋅03	1⋅88 ± 0⋅01	0⋅038
Riboflavin (mg/d)	1⋅82 ± 0⋅03	1⋅82 ± 0⋅02	1⋅90 ± 0⋅01	<0⋅0001
Pyridoxine (mg/d)	1⋅95 ± 0⋅03	1⋅98 ± 0⋅02	2⋅00 ± 0⋅01	0⋅187
Folate (mg/d)	565⋅39 ± 8⋅45	574⋅18 ± 5⋅88	582⋅41 ± 2⋅55	0⋅090
Cobalamin (μg/d)	2⋅96 ± 0⋅07	2⋅94 ± 0⋅05	2⋅98 ± 0⋅02	0⋅803
Long-chain omega-3 fatty acids (g/d)	1⋅62 ± 0⋅05	1⋅66 ± 0⋅03	1⋅78 ± 0⋅01	<0⋅0001
Caffeine (g/d)	117⋅48 ± 6⋅12	104⋅13 ± 4⋅26	96⋅32 ± 1⋅85	0⋅002
Food groups				
Fruits (g/d)	286⋅79 ± 15⋅87	294⋅45 ± 11⋅04	325⋅14 ± 4⋅79	0⋅005
Vegetables (g/d)	238⋅80 ± 8⋅12	239⋅30 ± 5⋅65	239⋅30 ± 2⋅45	0⋅998
Nuts, legumes and soy (g/d)	59⋅30 ± 2⋅50	56⋅62 ± 1⋅74	57⋅09 ± 0⋅75	0⋅659
White meat (g/d)	68⋅01 ± 3⋅04	63⋅11 ± 2⋅11	63⋅53 ± 0⋅92	0⋅348
Red meat (g/d)	81⋅53 ± 2⋅83	83⋅80 ± 1⋅97	77⋅40 ± 0⋅85	0⋅007
Refined grains (g/d)	380⋅63 ± 11⋅53	398⋅14 ± 8⋅03	396⋅35 ± 3⋅48	0⋅400
Whole grains (g/d)	45⋅15 ± 5⋅33	44⋅54 ± 3⋅71	43⋅37 ± 1⋅61	0⋅921

a Eating breakfast frequency was defined as seldom: never or 1 d/week; sometimes: 2–4 d/week and always: ≥5 d/week. The nutrients were adjusted for age, sex and energy. Values are mean ± se.

b Derived from ANCOVA.

Table 4 shows the crude and multivariable-adjusted ORs (95 % CI) for being in higher tertiles of psychological problems profile scores across the categories of breakfast consuming frequency. In the crude model, breakfast skipping was associated with higher risk of worse psychological problems (OR for seldom breakfast consumption 3⋅59; 95 % CI 2⋅52, 5⋅11; *P* < 0⋅0001). Adjustment for various confounders changed the odds very slightly, while in the final adjusted model, the OR and its corresponding 95 % for being in the highest tertile of psychological problems profile was 3⋅35 (2⋅11, 5⋅32; *P* < 0⋅0001) in the seldom category of breakfast eating.

**Table 4.** Multivariable-adjusted odds ratio and 95 % confidence interval for association of levels of psychological problems profiles scores across the categories of breakfast consuming frequency^a^

Psychological problems profile scores tertiles	Categories of breakfast consuming frequency			*P* trend
	Seldom (*n* 205)	Sometimes (*n* 424)	Always (*n* 2247)	
Crude model				<0⋅0001
Lowest tertile	1 (reference)	1 (reference)	1 (reference)	
Intermediate tertile	1⋅42 (0⋅96, 2⋅11)	1⋅18 (0⋅91, 1⋅55)	1 (reference)	
Highest tertile	3⋅59 (2⋅52, 5⋅11)	2⋅20 (1⋅72, 2⋅82)	1 (reference)	
Model 1				<0⋅0001
Lowest tertile	1 (reference)	1 (reference)	1 (reference)	
Intermediate tertile	1⋅57 (1⋅01, 2⋅46)	1⋅23 (0⋅92, 1⋅64)	1 (reference)	
Highest tertile	3⋅79 (2⋅52, 5⋅71)	2⋅33 (1⋅77, 3⋅08)	1 (reference)	
Model 2				<0⋅0001
Lowest tertile	1 (reference)	1 (reference)	1 (reference)	
Intermediate tertile	1⋅63 (1⋅02, 2⋅62)	1⋅22 (0⋅90, 1⋅65)	1 (reference)	
Highest tertile	4⋅07 (2⋅64, 6⋅26)	2⋅38 (1⋅78, 3⋅17)	1 (reference)	
Model 3				<0⋅0001
Lowest tertile	1 (reference)	1 (reference)	1 (reference)	
Intermediate tertile	1⋅55 (0⋅96, 2⋅51)	1⋅24 (0⋅91, 1⋅68)	1 (reference)	
Highest tertile	3⋅48 (2⋅20, 5⋅50)	2⋅37 (1⋅74, 3⋅22)	1 (reference)	
Model 4				<0⋅0001
Lowest tertile	1 (reference)	1 (reference)	1 (reference)	
Intermediate tertile	1⋅52 (0⋅94, 2⋅45)	1⋅18 (0⋅86, 1⋅61)	1 (reference)	
Highest tertile	3⋅35 (2⋅11, 5⋅32)	2⋅25 (1⋅65, 3⋅06)	1 (reference)	

a Multinomial logistic regression model was used.

Model 1: Adjustment was made for age, sex, marital status and education. Model 2: Additional adjustment was made for body mass index, smoking and physical activity. Model 3: Psychotropic medicines use and gastrointestinal disorders were additionally adjusted. Model 4: Additional adjustment for dietary intakes including magnesium, riboflavin, pyridoxine, folate, cobalamin, Docosahexaenoic acid and Eicosapentaenoic acid, energy, fibre and caffeine.

In stratified analyses by sex (Table 5), skipping breakfast was associated with a higher risk of being in the highest tertile of psychological problems profile scores in both men (OR 2⋅28; 95 % CI 1⋅39, 3⋅74; *P* = 0⋅004) and women (OR 2⋅26; 95 % CI 1⋅62, 3⋅14; *P* < 0⋅0001) in the crude model. These associations were not considerably influenced by adjusting the impacts of various covariates. In the final adjusted model, the odds for being in the highest tertile of psychological problems profile scores was 5⋅00 (95 % CI 2⋅29, 10⋅90; *P* < 0⋅0001) in men and 2⋅75 (95 % CI 1⋅55, 4⋅89; *P* < 0⋅0001) in women who ate breakfast seldom. We also observed an inverse significant correlation between frequency breakfast consumption per week and psychological problems profile scores either in men or in women (men: *r* −0⋅142 and in women: *r* −0⋅190; *P* < 0⋅0001). In the whole population, the corresponding value was −0⋅176 (*P* < 0⋅0001).

**Table 5.** Multivariable-adjusted odds ratio and 95 % confidence interval for association of levels of psychological problems profiles scores across the categories of breakfast consuming frequency by sex^a^

Psychological problems profile scores tertiles	Categories of breakfast consuming frequency			*P* trend
	Seldom (*n* 205)	Sometimes (*n* 424)	Always (*n* 2247)	
Men				
Crude model				<0⋅0001
Lowest tertile	1 (reference)	1 (reference)	1 (reference)	
Intermediate tertile	1⋅30 (0⋅68, 2⋅49)	1⋅26 (0⋅86, 1⋅84)	1 (reference)	
Highest tertile	3⋅93 (2⋅21, 6⋅98)	1⋅43 (0⋅94, 2⋅17)	1 (reference)	
Model 1				<0⋅0001
Lowest tertile	1 (reference)	1 (reference)	1 (reference)	
Intermediate tertile	1⋅56 (0⋅73, 3⋅31)	1⋅26 (0⋅82, 1⋅93)	1 (reference)	
Highest tertile	4⋅76 (2⋅43, 9⋅33)	1⋅65 (1⋅04, 2⋅63)	1 (reference)	
Model 2				<0⋅0001
Lowest tertile	1 (reference)	1 (reference)	1 (reference)	
Intermediate tertile	1⋅59 (0⋅71, 3⋅57)	1⋅28 (0⋅82, 2⋅00)	1 (reference)	
Highest tertile	5⋅28 (2⋅57, 10⋅84)	1⋅80 (1⋅12, 2⋅89)	1 (reference)	
Model 3				<0⋅0001
Lowest tertile	1 (reference)	1 (reference)	1 (reference)	
Intermediate tertile	1⋅50 (0⋅66, 3⋅41)	1⋅35 (0⋅86, 2⋅12)	1 (reference)	
Highest tertile	4⋅88 (2⋅26, 10⋅58)	2⋅02 (1⋅21, 3⋅37)	1 (reference)	
Model 4				<0⋅0001
Lowest tertile	1 (reference)	1 (reference)	1 (reference)	
Intermediate tertile	1⋅49 (0⋅66, 3⋅39)	1⋅31 (0⋅83, 2⋅07)	1 (reference)	
Highest tertile	5⋅00 (2⋅29, 10⋅90)	1⋅98 (1⋅18, 3⋅30)	1 (reference)	
Women				
Crude model				<0⋅0001
Lowest tertile	1 (reference)	1 (reference)	1 (reference)	
Intermediate tertile	1⋅40 (0⋅84, 2⋅32)	1⋅14 (0⋅78, 1⋅66)	1 (reference)	
Highest tertile	3⋅13 (1⋅99, 4⋅93)	2⋅62 (1⋅88, 3⋅67)	1 (reference)	
Model 1				<0⋅0001
Lowest tertile	1 (reference)	1 (reference)	1 (reference)	
Intermediate tertile	1⋅63 (0⋅93, 2⋅85)	1⋅23 (0⋅82, 1⋅84)	1 (reference)	
Highest tertile	3⋅36 (2⋅02, 5⋅61)	2⋅76 (1⋅92, 3⋅97)	1 (reference)	
Model 2				<0⋅0001
Lowest tertile	1 (reference)	1 (reference)	1 (reference)	
Intermediate tertile	1⋅66 (0⋅92, 2⋅99)	1⋅17 (0⋅77, 1⋅79)	1 (reference)	
Highest tertile	3⋅53 (2⋅06, 6⋅04)	2⋅72 (1⋅86, 3⋅96)	1 (reference)	
Model 3				<0⋅0001
Lowest tertile	1 (reference)	1 (reference)	1 (reference)	
Intermediate tertile	1⋅57 (0⋅87, 2⋅86)	1⋅15 (0⋅75, 1⋅75)	1 (reference)	
Highest tertile	2⋅97 (1⋅68, 5⋅22)	2⋅55 (1⋅71, 3⋅80)	1 (reference)	
Model 4				<0⋅0001
Lowest tertile	1 (reference)	1 (reference)	1 (reference)	
Intermediate tertile	1⋅47 (0⋅80, 2⋅68)	1⋅06 (0⋅69, 1⋅62)	1 (reference)	
Highest tertile	2⋅75 (1⋅55, 4⋅89)	2⋅36 (1⋅58, 3⋅53)	1 (reference)	

a Multinomial logistic regression model was used.

Model 1: Age, marital status and education were adjusted. Model 2: Additional adjustments were done for weight, smoking and physical activity. Model 3: Further controls were done for psychotropic medicines use and gastrointestinal disorders were made Model 4: Additional adjustment for dietary intakes including magnesium, riboflavin, pyridoxine, folate, cobalamin, Docosahexaenoic acid and Eicosapentaenoic acid, energy, fibre and caffeine.

The results of the association between DII/breakfast habit (interaction of breakfast skipping and DII levels) and psychological problems profile scores in the whole population and separately in men and women are presented in Table 6. In the crude multinomial logistic regression model, participants with high DII (>median) who did not consume breakfast had higher risk of being in highest tertile of psychological problems profile scores (OR 4⋅71; 95 % CI 2⋅94, 7⋅55) and after adjustment for potential confounder the association strengthen (OR 6⋅67; 95 % CI 3⋅45, 12⋅90). Also, who skipped breakfast with either high or low DII had an increased risk of being in the higher tertiles of psychological problems profile compared with those who had low DII (<median) and ate breakfast. Although adjustment for potential confounders slightly changed the associations, however they remained statistically significant. In the stratified analysis by sex, findings in both men and women were consistent with the whole population.

**Table 6.** Multivariable-adjusted odds ratio and 95 % confidence interval for association between DII/breakfast habit and psychological problems profile in the whole population and separately in men and women

Psychological problems profile scores tertiles	Psychological problems profile scores tertiles			*P* trend
	1 (*n* 958)	2 (*n* 959)	3 (*n* 959)	
Whole population				
Crude model				<0⋅0001
Low DII/eat breakfast	1 (reference)	1 (reference)	1 (reference)	
High DII/ skip breakfast	1 (reference)	1⋅53 (0⋅86, 2⋅73)	4⋅71 (2⋅94, 7⋅55)	
Low DII/ skip breakfast	1 (reference)	1⋅48 (0⋅86, 2⋅55)	2⋅98 (1⋅75, 5⋅08)	
High DII/eat breakfast	1 (reference)	1⋅18 (0⋅99, 1⋅41)	1⋅53 (1⋅28, 1⋅83)	
Multivariable-adjusted model^a^				<0⋅0001
Low DII/eat breakfast	1 (reference)	1 (reference)	1 (reference)	
High DII/skip breakfast	1 (reference)	1⋅76 (0⋅86, 3⋅59)	6⋅67 (3⋅45, 12⋅90)	
Low DII/skip breakfast	1 (reference)	1⋅29 (0⋅63, 2⋅65)	2⋅23 (1⋅10, 4⋅53)	
High DII/eat breakfast	1 (reference)	1⋅34 (1⋅07, 1⋅65)	1⋅80 (1⋅41, 2⋅30)	
Men				
Crude model				<0⋅0001
Low DII/eat breakfast	1 (reference)	1 (reference)	1 (reference)	
High DII/skip breakfast	1 (reference)	1⋅49 (0⋅45, 4⋅95)	6⋅23 (2⋅17, 17⋅89)	
Low DII/skip breakfast	1 (reference)	1⋅15 (0⋅53, 2⋅51)	4⋅10 (2⋅05, 8⋅23)	
High DII/eat breakfast	1 (reference)	0⋅97 (0⋅75, 1⋅25)	1⋅49 (1⋅11, 2⋅01)	
Multivariable-adjusted model^a^				<0⋅0001
Low DII/eat breakfast	1 (reference)	1 (reference)	1 (reference)	
High DII/skip breakfast	1 (reference)	3⋅23 (0⋅60⋅17⋅29)	6⋅47 (2⋅45, 17⋅13)	
Low DII/skip breakfast	1 (reference)	1⋅12 (0⋅38, 3⋅34)	1⋅58 (1⋅08, 2⋅23)	
High DII/ eat breakfast	1 (reference)	1⋅11 (0⋅80, 1⋅55)	1⋅70 (1⋅12, 2⋅57)	
Women				
Crude model				<0⋅0001
Low DII/eat breakfast	1 (reference)	1 (reference)	1 (reference)	
High DII/skip breakfast	1 (reference)	1⋅93 (0⋅88, 4⋅22)	5⋅78 (2⋅90, 11⋅52)	
Low DII/skip breakfast	1 (reference)	1⋅48 (1⋅16, 1⋅90)	2⋅02 (1⋅09, 3⋅75)	
High DII/eat breakfast	1 (reference)	1⋅46 (0⋅75, 2⋅82)	1⋅78 (1⋅40, 2⋅27)	
Multivariable-adjusted model^a^				<0⋅0001
Low DII/eat breakfast	1 (reference)	1 (reference)	1 (reference)	
High DII/ skip breakfast	1 (reference)	2⋅53 (0⋅92, 6⋅91)	2⋅58 (1⋅02, 6⋅58)	
Low DII/skip breakfast	1 (reference)	1⋅58 (1⋅17, 2⋅13)	1⋅97 (1⋅44, 2⋅69)	
High DII/eat breakfast	1 (reference)	1⋅07 (0⋅48, 2⋅42)	1⋅88 (1⋅34, 2⋅52)	

a Adjustment was made for age, gender (not in stratified analyses by gender) marital status and education, weight, smoking and physical activity, psychotropic medicines use and gastrointestinal disorders

## Discussion

The present study found that breakfast skipping is associated with increased risk of higher scores of psychological problems profile in a general population of Iranian adults. Also, our analyses showed that the breakfast skipping in combination with low diet quality, determined by DII, more adversely affect the psychological health; in which we observed that those participants who skipped breakfast and ate diet with higher pro-inflammatory content had highest risk of larger psychological problems profile scores. In the stratified analysis by sex, this direct link was observed among both men and women, but much stronger among men than women.

Due to differences in socioeconomic status, lifestyle and dietary habits among countries, it is conceivable that findings derived from observational studies may vary between countries. In Iran, a relatively large fraction of total years of life lived with disability (YLD) is caused by mental disorders^(54)^; and therefore, identifying the risk factors contributing in the high prevalence of mental disorders among Iranians is relevant.

To date, the association between breakfast eating and psychological health, violent behaviours and happiness has been evaluated among Iranian children, adolescents and college students^(55,56)^. However, the largest proportion of YLDs from mental disorders in Iranians occurs in young adults (19–39 years)^(54)^; and therefore, taking into account the differences in lifestyle between adults and children/adolescents, it would be worthwhile to evaluate such association among young adults. To our knowledge, there is only one study in Iranian adults which assessed the breakfast-mental disorders association^(57)^. In the present study, because of high incidence of comorbid or co-occurring of psychological problems^(58)^, we constructed a score providing a comprehensive overview for the mental health status of participants based on applying sophisticated statistical method, i.e. factor analysis through combining the scores of three common mental health disorders (depression, anxiety and psychological distress). In line with our previous analysis^(57)^, which indicated an indirect link between breakfast consumption and depression, anxiety, and psychological distress risk^(57)^, the newly constructed model also confirmed the beneficial association between breakfast consumption and mental health status. Moreover, in the present study, we evaluated the role of diet quality, determined by the inflammatory potential of diet, intricately with breakfast skipping on psychological health. Taken together, a number of dietary components have been related to pro- or anti-inflammatory pathways and the circulating concentrations of pro- or anti-inflammatory mediators. Despite this, it has been suggested that taking into account diet as a whole, in combination with dietary habits, may be important, because of the role of potential interactions between, and/or synergistic effects of, food and habits components.

The findings from previous studies regarding the association of breakfast skipping with mental disorders are inconsistent. A longitudinal study among Japanese workers, who were high risk for depressive symptoms, has indicated no beneficial effect of breakfast eating on depression incidence after a 2-year follow-up, while the baseline results of this study confirmed the direct link between breakfast skipping and depressive symptoms^(12)^. In contrast, Korean Community Health Survey provided evidence that individuals who had breakfast seldom or sometimes had higher risk for depression compared with individuals who had breakfast always^(11)^. Consistently, another cross-sectional field study suggested that older adults who received home-delivered breakfast for 5 d in a week had fewer depressive symptoms^(13)^. In the secondary school children, breakfast skipping was associated with greater risk of distress, anxiety and depression in a cross-sectional design, while following children for 6 months revealed no significant link^(18)^. A direct link between breakfast skipping and psychological distress has been also suggested in two other cross-sectional studies^(17,19)^. Differences in study design, study population and methods of depression assessment may contribute to the discrepancies in the results of studies. Although based on available evidence and our results, breakfast skipping is cross-sectionally related to the risk of higher scores of psychological problems, it is impossible to determine whether breakfast skipping is a cause or a result of affecting by psychological disorders using cross-sectional analysis. For example, taking psychotropic medicines may disrupt sleep cycles and consequently influence eating behaviours, and interests in daily activities such as eating. In addition, the poverty of participants may lead to skipping meals or the feeling of being cared for/not isolated in elderlies who received home-delivered breakfast may be the reason for fewer depressive symptoms. Therefore, drawing a conclusion in this regard should be done cautiously.

We observed a stronger association between breakfast skipping and breakfast skipping/DII interaction with higher scores of psychological problems profile in men than women in stratified analyses. The reasons for this discrepancy are not clear and require further investigations. However, it is possible that the stronger association among men may be due to different personality traits^(59)^ and sex differences in the use of coping strategies^(60)^ which seems to be involved in the pathogenesis of anxiety and depression^(61)^. It has been shown that in women, using more positive reframing was associated with lower depressive symptoms, while in men, depressive symptoms were independent from positive reframing^(60)^.

The exact mechanisms underlying the favourable effect of breakfast eating on moods have not been elucidated. It is probable that breakfast eaters have better mental health status because of having a healthier lifestyle. Nevertheless, we observed that even after controlling for lifestyle factors, including physical activity, dietary intakes and smoking, breakfast skippers had higher risk of higher scores of psychological problems. Another possible explanation might be higher levels of cortisol in breakfast skippers which stimulates hypothalamic–pituitary–adrenal (HPA) axis independent of stress^(25)^. Over-activity of the HPA axis has been suggested as a plausible mechanism in all depressive disorders^(62)^. Furthermore, elevated levels of cortisol are associated with greater inflammation which contributes to mental disorders particularly depression by affecting neurotransmitters synthesis^(63)^. Consistently, our analysis supports the interrelationship of DII and breakfast eating habit with psychological problems profile which is in line with other investigations indicating a positive association between DII and mental disorders^(64)^. Although it is not clear which inflammatory mediators, and to which extent, contribute to developing mental disorders, however, previous studies suggest that diet-related inflammatory effects may be mediated via mediators other than serum inflammatory biomarkers such as IL-6 and C-Reactive Protein (CRP)^(31,65)^. Moreover, since CRP and IL-6 levels are affected by many modifying factors, such as age, adiposity, physical fitness, activity and dietary habits, they are unlikely specific indicators for systematic inflammation *per se*^(65)^. Thus, further research investigating the link between DII and inflammatory mediators such as dietary habits including meals consumption is required.

The present study has several limitations which should be taken into account when interpreting the results. First, the study population was not truly a representative sample of the whole Iranian population. Nevertheless, this would be more important in a descriptive report than in an analytical study like this. Moreover, because of the large sample size and good distribution of potential confounders, it is unlikely that our findings are affected by the lack of representativeness. Second, since this study was a cross-sectional, we could not draw any causal inferences. For example, psychological problems may reduce appetite and lead to breakfast skipping, an association that cannot be evaluated using the present data. Third, both exposure and outcome were collected using self-administered questionnaires in this study. Applying more sophisticated tools to assess psychological problems may lead to stronger associations. Fourth, despite controlling for various confounders, there may be some unknown or unmeasured confounders which cannot be excluded. For instance, sleep quality and duration, wake up time, and bedtime may affect both breakfast skipping and mental health as well as nutrients intake. Finally, although turning continuous variables into categorical variables is not a statistically preferred approach, we used the tertiles of psychological problems profile to simplify the clinical interpretations of mental disorders as well as the presentation of results which are the common method in epidemiological studies^(66)^. Substantially large sample size and considering the confounding effects of various variables are the strengths of the present study.

In conclusion, our findings confirm the previous evidence, suggesting that breakfast skipping is associated with higher risk of psychological problems in a general population of Iranian adults. Moreover, our further analysis confirmed that the DII and breakfast skipping are associated with mental health, interactionally. This association was not explained by some measures like socioeconomic status, physical activity, smoking and dietary intakes. Therefore, eating breakfast could be a simple approach to promote mental health among Iranians, especially men. Further longitudinal studies are needed to confirm the true link between breakfast skipping and psychological problems and to investigate possible mechanisms.

## Supplementary material

For supplementary material accompanying this paper visit https://doi.org/10.1017/jns.2020.62. click here to view supplementary material
