# Supplementary material for: Breakfast skipping alone and in interaction with inflammatory based quality of diet increases the risk of higher scores of psychological problems profile in a large sample of Iranian adults
Source: J Nutr Sci. 2021 Feb 16;10:e10. doi: 10.1017/jns.2020.62 (PMC8057508; doi:10.1017/jns.2020.62)
Supplement: Supplementary file 1 [file S2048679020000622sup001.docx]

Table 1- General characteristics of participants across categories of breakfast consuming frequency stratified by sex^1^.

| **Variables** | **Breakfast consuming frequency (Men)** | | | | **Breakfast consuming frequency (women)** | | | |
| --- | --- | --- | --- | --- | --- | --- | --- | --- |
|  | **Seldom** | **Sometimes** | **Always** | **P value^2^** | **Seldom** | **Sometimes** | **Always** | **P value^2^** |
| Participants (n) | 205 | 424 | 2247 |  | 205 | 424 | 2247 |  |
| Age (y)^3^ | 35.8±8.6 | 37.5±8.1 | 37.7±8.0 | <0.0001 | 34.7±7.2 | 33.6±7.2 | 33.3±7.0 | <0.0001 |
| BMI (kg/m^2^)^3^ | 25.7±3.8 | 25.6±3.5 | 25.3±3.4 | 0.270 | 24.4±3.9 | 24.8±4.4 | 24.5±4.0 | 0.675 |
| Depression score | 6.9±4.0 | 5.7±3.2 | 5.3±2.9 | <0.0001 | 7.6±3.3 | 7.5±3.6 | 6.3±3.1 | <0.0001 |
| Anxiety score | 4.4±4.5 | 3.2±3.6 | 2.8±3.0 | <0.0001 | 5.3±4.3 | 5.2±4.2 | 3.7±3.4 | <0.0001 |
| Psychological distress score | 3.3±3.4 | 1.6±2.2 | 1.8±2.2 | <0.0001 | 3.3±3.4 | 3.1±3.1 | 3.7±3.4 | <0.0001 |
| Depressed (%) | 35.5 | 22.2 | 19.4 | 0.004 | 49.1 | 44.6 | 29.9 | <0.0001 |
| Anxious (%) | 18.2 | 9.9 | 7.5 | 0.004 | 24.8 | 27.0 | 14.0 | <0.0001 |
| Psychologically distressed (%) | 38.2 | 15.1 | 16.2 | <0.0001 | 40.6 | 40.0 | 23.4 | <0.0001 |
| Marital status (%) |  |  |  | 0.408 |  |  |  | 0.518 |
| Married | 86.7 | 90.8 | 89.8 |  | 76.6 | 78.2 | 75.2 |  |
| Single | 12.0 | 9.2 | 9.9 |  | 22.2 | 19.0 | 22.2 |  |
| Other | 1.3 | 0.0 | 0.3 |  | 1.3 | 2.8 | 2.6 |  |
| Psychotropic medicines (%) | 7.8 | 3.4 | 3.5 | 0.288 | 15.5 | 8.5 | 5.7 | <0.0001 |
| Current smokers (%) | 7.2 | 10.1 | 6.5 | 0.407 | 0.7 | 0.4 | 0.0 | 0.315 |
| Physical activity >1 h/wk (%) | 48.6 | 42.2 | 42.9 | 0.716 | 21.2 | 23.2 | 27.8 | 0.140 |
| Educational level |  |  |  | 0.402 |  |  |  | 0.026 |
| <12 y | 41.3 | 50.6 | 50.4 |  | 42.6 | 29.3 | 29.8 |  |
| 12-16 y | 44.0 | 38.9 | 40.1 |  | 52.9 | 66.2 | 63.3 |  |
| ≥16 y | 14.7 | 10.5 | 8.8 |  | 4.5 | 4.6 | 6.9 |  |
| FGID^4^ (%) | 67.5 | 61.8 | 59.0 | 0.455 | 75.8 | 76.9 | 71.1 | 0.002 |
| IBS (%) | 28.6 | 14.6 | 18.3 | 0.069 | 24.8 | 24.7 | 25.0 | 0.025 |
| Functional dyspepsia (%) | 16.9 | 11.8 | 11.1 | 0.457 | 23.0 | 20.3 | 15.0 | 0.010 |
| [Gastroesophageal Reflux Disease](https://www.ncbi.nlm.nih.gov/pubmed/31049179) (%) | 27.3 | 21.3 | 21.4 | 0.197 | 36.0 | 30.5 | 22.2 | <0.0001 |
| Functional bloating (%) | 15.6 | 24.7 | 21.9 | 0.248 | 16.8 | 20.7 | 19.9 | 0.783 |
| Constipation (%) | 36.4 | 28.7 | 23.2 | 0.028 | 47.2 | 46.4 | 38.2 | <0.0001 |
| High DII (More than median of DII score) (%) | 37.5 | 51.7 | 52.3 | 0.065 | 54.7 | 52.2 | 47.0 | 0.038 |
| DII and breakfast eating habit |  |  |  | <0.0001 |  |  |  | <0.0001 |
| Low DII & eat breakfast (%) | 0.0 | 48.3 | 47.7 |  | 0.0 | 47.8 | 53.0 |  |
| High DII & eat breakfast (%) | 0.0 | 51.7 | 52.3 |  | 0.0 | 52.2 | 47.0 |  |
| Low DII & skip breakfast (%) | 32.5 | 0.0 | 0.0 |  | 45.3 | 0.0 | 0.0 |  |
| High DII & skip breakfast (%) | 67.5 | 0.0 | 0.0 |  | 54.7 | 0.0 | 0.0 |  |

BMI: body mass index; FGID: functional gastrointestinal disorders; IBS: rritable bowel syndrome; DII: dietary inflammatory index.

^1^Eating breakfast frequency was defined as seldom: never or one day/wk, sometimes: 2-4 days/wk and always: ≥5 days/wk.

^2^Derived from one-way ANOVA and chi-square test for continuous and categorical variables, respectively.^3^Values are means±SEs.

^4^FGID defined as suffering from at least one of the following disorders: [gastroesophageal reflux](https://www.ncbi.nlm.nih.gov/pubmed/28405322), dyspepsia, irritable bowel syndrome and constipation.

Table 2- General characteristics across tertiles of psychological problems profile scores stratified by sex^1^. (Mean±SD)

| **Variables** | **Tertiles of psychological problems profile scores (Men)** | | | | **Tertiles of psychological problems profile scores (women)** | | | |
| --- | --- | --- | --- | --- | --- | --- | --- | --- |
|  | **1(n=958)** | **2(n=959)** | **3(n=959)** | **P value^2^** | **1(n=958)** | **2(n=959)** | **3(n=959)** | **P value^2^** |
| Age (years) | 37.1±8.2 | 39.4±8.3 | 38.3±7.8 | <0.0001 | 34.7±7.1 | 34.8±7.4 | 34.9±7.2 | 0.817 |
| BMI (kg/m^2^) | 25.3±3.2 | 25.4±3.6 | 25.3±3.7 | 0.781 | 24.5±3.9 | 24.4±3.8 | 24.6±4.2 | 0.542 |
| Depression score | 3.0±1.4 | 5.6±1.5 | 9.3±3.0 | <0.0001 | 3.2±1.3 | 5.7±1.4 | 9.6±2.9 | <0.0001 |
| Anxiety score | 0.6±0.8 | 2.6±1.5 | 7.0±3.7 | <0.0001 | 0.7±0.9 | 2.6±1.5 | 7.4±3.7 | <0.0001 |
| Psychological distress score | 0.2±0.5 | 1.2±1.3 | 4.7±2.9 | <0.0001 | 0.2±0.5 | 1.2±1.2 | 4.8±2.9 | <0.0001 |
| Depressed (%) | 0.0 | 9.3 | 73.5 | <0.0001 | 0.0 | 11.3 | 76.2 | <0.0001 |
| Anxious (%) | 0.0 | 0.2 | 35.5 | <0.0001 | 0.0 | 0.2 | 42.9 | <0.0001 |
| Psychological distressed (%) | 0.0 | 6.0 | 63.6 | <0.0001 | 0.0 | 5.6 | 64.3 | <0.0001 |
| Married (%) | 87.8 | 93.0 | 89.2 | 0.087 | 73.4 | 75.3 | 76.9 | 0.034 |
| Educational level (%) |  |  |  | 0.402 |  |  |  | 0.026 |
| ≤12 yr | 41.3 | 50.6 | 50.4 |  | 28.5 | 29.3 | 29.8 |  |
| 12-16 yr | 44.0 | 38.9 | 40.6 |  | 52.9 | 66.2 | 42.5 |  |
| >16 yr | 14.7 | 10.5 | 8.8 |  | 4.5 | 4.6 | 6.9 |  |
| Physical activity >1 h/wk (%) | 48.9 | 40.8 | 36.7 | 0.002 | 31.2 | 26.4 | 23.2 | 0.010 |
| Psychotropic medicines use^4^ (%) | 2.0 | 2.0 | 9.3 | <0.0001 | 1.9 | 4.1 | 12.5 | <0.0001 |
| FGID^3^ (yes) (%) | 43.1 | 67.4 | 81.9 | <0.0001 | 51.1 | 73.4 | 86.7 | <0.0001 |
| IBS (%) | 9.8 | 16.9 | 35.5 | <0.0001 | 10.8 | 23.6 | 36.2 | <0.0001 |
| Functional dyspepsia (%) | 2.9 | 11.8 | 27.7 | <0.0001 | 4.6 | 13.9 | 27.1 | <0.0001 |
| [Gastroesophageal Reflux Disease](https://www.ncbi.nlm.nih.gov/pubmed/31049179) (%) | 13.6 | 22.8 | 36.1 | <0.0001 | 12.5 | 21.0 | 36.2 | <0.0001 |
| Functional bloating (%) | 21.8 | 27.3 | 18.4 | 0.011 | 19.3 | 22.0 | 17.4 | 0.104 |
| Constipation (%) | 10.4 | 26.4 | 46.4 | <0.0001 | 23.3 | 39.3 | 53.2 | <0.0001 |
| Breakfast skippers (%) | 3.5 | 4.4 | 11.9 | <0.0001 | 5.2 | 7.0 | 12.6 | <0.0001 |
| High DII (More than median of DII score) (%) | 50.6 | 50.1 | 59.8 | 0.013 | 39.7 | 48.7 | 54.6 | <0.0001 |
| Current smokers (%) | 5.3 | 6.5 | 10.7 | 0.012 | 0.0 | 0.4 | 0.2 | 0.388 |
| DII and breakfast eating habit |  |  |  | <0.0001 |  |  |  | <0.0001 |
| Low DII & eat breakfast (%) | 48.1 | 48.4 | 35.5 |  | 57.3 | 47.2 | 40.3 |  |
| High DII & eat breakfast (%) | 48.4 | 47.2 | 52.9 |  | 37.6 | 45.8 | 47.1 |  |
| Low DII & skip breakfast (%) | 0.9 | 1.4 | 4.2 |  | 3.2 | 3.8 | 4.5 |  |
| High DII & skip breakfast (%) | 2.6 | 3.0 | 7.7 |  | 2. | 3.2 | 8.1 |  |

BMI: body mass index; FGID: functional gastrointestinal disorders; IBS: rritable bowel syndrome; DII: dietary inflammatory index.

^1^ Values are Mean±SE unless otherwise indicated. BMI: body mass index. FGID: functional gastrointestinal disorders.

^2^ Resulted from one-way ANOVA and χ^2^ test for continuous and categorical variables, respectively.

^3^ FGID defined as suffering from at least one of the following gastrointestinal disorders: gastroesophageal reflux, dyspepsia, irritable bowel syndrome and constipation. Overweight was defined as BMI greater than or equal to 25 and less than or equal to 29.99 kg/m^2^ and obese was defined as BMI≥30 kg/m^2^.
